# Prediction of improved survival in patients with pancreatic cancer via IL-21 enhanced detection of mesothelin epitope-reactive T-cell responses

**DOI:** 10.18632/oncotarget.25121

**Published:** 2018-04-27

**Authors:** Qingda Meng, Davide Valentini, Martin Rao, Zhenjiang Liu, Shanshan Xie, Ann Morgell, Ernest Dodoo, Matthias Löhr, Elena Rangelova, Marco del Chiaro, Ingemar Ernberg, Markus Maeurer

**Affiliations:** ^1^ Division of Therapeutic Immunology (TIM), Department of Laboratory Medicine (LABMED), Karolinska Institutet, Stockholm, Sweden; ^2^ Centre for Allogeneic Stem Cell Transplantation (CAST), Karolinska University Hospital Huddinge, Stockholm, Sweden; ^3^ Pancreatic Surgery Unit, Division of Surgery, Department of Clinical Science, Intervention and Technology (CLINTEC), Karolinska Institutet, Stockholm, Sweden; ^4^ Department of Microbiology, Tumor and Cell Biology, Karolinska Institutet, Stockholm, Sweden

**Keywords:** pancreatic cancer, mesothelin, interferon gamma, survival, antigen-specific response

## Abstract

Most patients with pancreatic cancer present with extensive metastasis at diagnosis, with a 5-year survival rate of approximately 5%, despite chemotherapy and surgery. New treatment modalities are needed to improve survival. Mesothelin is a tumor-associated antigen (TAA) in patients with pancreatic cancer that could be used to gauge cellular immune responses directed against transformed cells since up to 100 percent of pancreatic ductal adenocarcinoma cells have been shown to strongly express mesothelin. A prospective, observational study was carried out in twenty-six, chemotherapy-naïve patients with resectable pancreatic ductal adenocarcinoma. Participants were between 48 and 81 years (median age: 64.5 years), 15 males and 11 females. All participants were clinically followed-up between 439 and 853 days post-surgery (n=14) or until death (n=12). Peripheral blood drawn on the day of surgery was stimulated with a mesothelin peptide pool (42 peptides, non-overlapping), individual mesothelin peptides, positive (anti-CD3 antibody, OKT3) and negative controls (medium) with or without adding IL-21. Kaplan-Meier estimators were used to gauge patients’ survival pattern in relation to mesothelin-specific IFN-γ responses. A survival benefit was linked with IFN-γ responses to peptides corresponding to mature mesothelin (p=0.018) and targeted recognition of the mesothelin_601-615_ epitope (MQEALSGTPCLLGPG) (p=0.006) in the presence of IL-21. Conversely, production of high levels of IFN-γ to OKT3 stimulation with IL-21 conditioning was associated with reduced survival of patients (p=0.016). Gauging anti-Mesothelin- directed immune responses will aid to identify patients i) in need of a more intensive clinical follow-up and ii) who may benefit from immunotherapeutic approaches targeting mesothelin.

## INTRODUCTION

Pancreatic cancer ranks as the 7^th^ most common human cancer, with a 5-year survival rate of 5% encompassing all stages of disease [1]. In 2012, 338,000 new cases were reported, while 330,000 deaths occurred despite chemotherapy, with or without surgery [1]. Europe and North America account for more than 40% of the worldwide burden of pancreatic cancer disease and mortality [1]. More than 90 % of newly diagnosed cases with pancreatic cancer represent ductal adenocarcinoma [1]. Only 1/5 of patients (20% of cases) qualify for surgery; the majority of patients present with metastatic disease at the time of diagnosis [2]. Improved treatment modalities for patients with pancreatic cancer are therefore urgently needed.

Current T cell-based immunotherapies have shown clinically durable responses not only in patients with melanoma, yet more recently also in patients with tumors of different histologies, associated with the activation and mobilization of T cells recognizing tumor-associated antigens (TAAs) [3]. Since not all TAAs elicit an equally efficacious immune response, novel targets that can mediate biologically and clinically relevant cellular immune responses are needed. Importantly, T cells targeting the patient's ‘private antigens’ i.e. tumor-associated mutations, including tumor ‘driver’ mutations such as V-Ki-ras2 Kirsten rat sarcoma viral oncogene homolog (KRAS) genetic aberrations, are associated with tumor regression and long-term survival in patients with gastrointestinal tumors [4].

Mature mesothelin represents a non-mutated, cell surface-bound TAA that is expressed by mesothelial cells lining various internal organs, including the pleura, pancreas, peritoneum and the pericard [5]. Overexpression of mesothelin has been identified in a number of human cancers e.g mesothelioma, lung cancer, colorectal cancer, ovarian cancer as well as pancreatic cancer [5–9]. Virtually all pancreatic adenocarcinomas/ductal adenocarcinomas have been found to be positive for mesothelin expression [10–12]. Mesothelin comprises several components: a 36-amino acid (aa)-long signal peptide, (i) the megakaryocyte-potentiating factor (MPF), which is shed into the bloodstream upon enzymatic cleavage (aa 37-286) and (ii) the GPI-anchored mature mesothelin, which is overexpressed in tumor tissues (aa 296-606; with a pro-peptide, aa 607-630, that is cleaved during maturation/activation) [5]. Mesothelin-specific T cell responses have been observed in peripheral blood lymphocytes from patients with pancreatic cancer, including individuals who were administered a cancer vaccine candidate overexpressing mesothelin peptides and granulocyte-macrophage colony-stimulating factor [6, 13–15]. Here we show that mesothelin-specific interferon gamma (IFN-γ) production by peripheral blood lymphocytes is a reliable predictor of survival among patients with pancreatic cancer. We also provide evidence that mesothelin-specific cellular immune responses can be amplified *in vitro* with interleukin (IL)-21 conditioning, possibly with preferential expansion of T-cells directed against TAAs. This is the first report to establish a clinically relevant link between mesothelin-directed cellular immune responses in peripheral blood lymphocytes and increased survival of patients with pancreatic cancer. Mesothelin-specific immune responses may also aid to identify patients i) at increased risk for tumor progression ii) who may benefit from a more frequent followup and alternate treatment strategies, including immunotherapeutic strategies with the aim to expand anti-cancer T-cells.

## RESULTS

**Figure 1:**
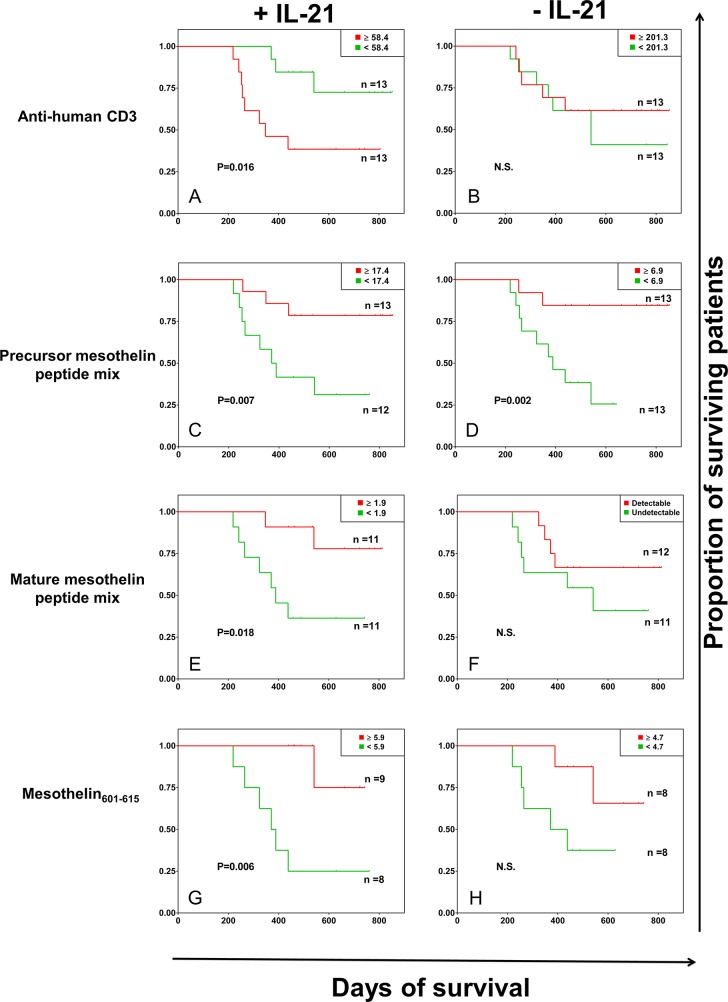
Kaplan-Meier survival analysis of patients with pancreatic cancer based on antigen-specific IFN-γ responses A whole blood assay was performed (7-day incubation) in 96-well microtiter plates, with or without IL-21 conditioning. The following stimulation conditions were applied: **(A and B)** anti-human CD3 antibody/OKT3 as positive control; **(C and D)** full-length mesothelin, the 42 peptides corresponding to the MPF and the mature mesothelin components; **(E and F)** mature mesothelin peptide mix, the 23 peptides spanning the GPI-anchored, cell surface-bound mature component of the mesothelin molecule –expressed on cancer cells; **(G and H)** mesothelin_601-615_, which was found to be highly immunogenic in an initial screen (presented in Supplementary Table 2 and Supplementary Figure 1). Culture medium was used as negative control. Supernatants were harvested for IFN-γ measurement by a standard sandwich ELISA, and the negative control values were subtracted from the final values reported. Shown are the survival of patients with pancreatic cancer in relation to IFN-γ production by peripheral blood T cells, based on the median cut-off values of IFN-γ levels (in pg/ml of cytokine concentration). P<0.05 was considered significant, while ‘N.S.’ denotes a non-significant p value.

A description of the patient cohort is provided in Table 1. Whole blood assays were used for gauging immune responses, since peripheral blood mononuclear cells (PBMCs) constitute a reliable readout of cellular immune responses. IL-21 has been shown to promote preferential expansion of high-affinity, antigen-specific T cells and to enrich antigen-experienced (memory) CD8+ and CD4+ T cells in the host [16–18]. These effects are also observed in association with IL-15 [19] as well as IL-7 that promote antigen-specific T-cell proliferation and anti-tumor T-cell functions [20]. In addition, we have previously reported a cytokine cocktail, consisting of IL-2, IL-15 and IL-21, that leads to the accumulation of central memory tumor infiltrating T-cells (TILs), obtained from patients with glioblastoma as well as pancreatic tumors [21, 22]. Therefore, we decided to use IL-21 in the experiments in order to investigate the ‘conditioning effect’ of IL-21 on mesothelin-specific T cells from patients with pancreatic cancer. Pertaining to evaluation of the patients’ cellular immune responses, strong IFN-γ production in response to anti-human CD3 antibody (OKT3) stimulation in peripheral blood from patients with pancreatic cancer appeared to be associated with a survival benefit in the presence, but not in the absence of IL-21 conditioning (Figure 1A & 1B). While robust IFN-γ responses to OKT3 without IL-21 conditioning did not correlate with patient survival, the patients whose peripheral blood lymphocytes produced more IFN-γ after OKT3 stimulation (with IL-21 conditioning) displayed a *negative* survival benefit (p=0.016). In contrast, increased IFN-γ responses to the precursor mesothelin peptide pool stimulation, with IL-21 conditioning, was associated with increased survival (p=0.007) (Figure 1C). A survival benefit in relation to precursor mesothelin-directed IFN-γ production was also observed when the patients’ peripheral blood was not conditioned with IL-21 (p=0.002) (Figure 1D). The positive correlation between survival and the precursor mesothelin-induced cellular immune response proved to be linked to the 23 peptides belonging to the cell-bound, mature mesothelin component (p=0.018) only in the presence of IL-21 conditioning (Figure 1E and 1F). We did not find an association between IFN-γ responses to the mesothelin MPF component and improved survival of patients with pancreatic cancer (data not shown).

**Table 1:** Clinical characteristics of participating patients

Patient ID (#)	Age (years)	Gender (M/F)	Diagnosis
2	68	M	Adenocarcinoma
5	74	M	Adenocarcinoma
7	71	M	Ductal adenocarcinoma
19	62	F	Ductal adenocarcinoma
24	81	M	Ductal adenocarcinoma
31	77	M	Ductal adenocarcinoma
34	50	M	Ductal adenocarcinoma
41	75	F	Ductal adenocarcinoma
50	75	F	Ductal adenocarcinoma
51	58	F	Ductal adenocarcinoma
59	60	M	Ductal adenocarcinoma
62	50	M	Ductal adenocarcinoma
63	61	M	Ductal adenocarcinoma
68	56	F	Ductal adenocarcinoma
72	48	F	Ductal adenocarcinoma
74	55	F	Ductal adenocarcinoma
76	54	M	Ductal adenocarcinoma
81	63	M	Ductal adenocarcinoma + BD-IPMN
87	66	F	Pancreatobiliary ductal adenocarcinoma
99	50	F	Pancreatobiliary ductal adenocarcinoma
104	76	F	Pancreatobiliary ductal adenocarcinoma
107	81	M	Pancreatobiliary ductal adenocarcinoma
114	62	F	Pancreatobiliary ductal adenocarcinoma
116	67	M	Pancreatobiliary ductal adenocarcinoma
117	70	M	Pancreatobiliary ductal adenocarcinoma
120	72	M	Pancreatobiliary ductal adenocarcinoma
**Age median**		64.5 years	
**Male:female ratio**	1.36:1	(M = 15, F = 11)	

**Figure 2:**
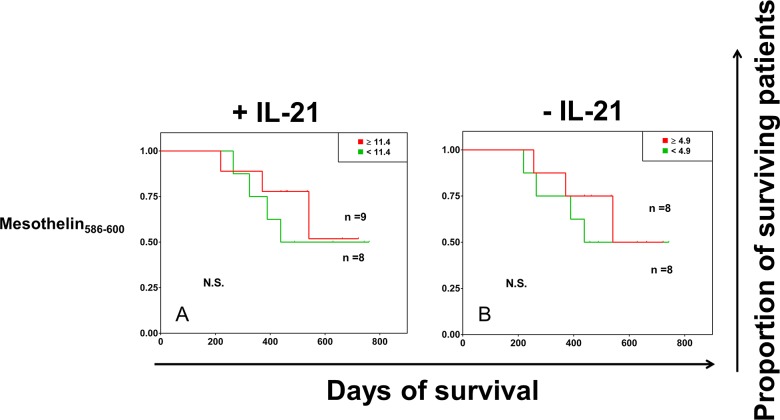
**(A and B)**. Identical experimental layout as in Figure 1. The peptide derived from mesothelin_586-600_ was used as a control peptide which was found to be recognized in the ‘hotspot identification’ (Supplementary Table 2 and Supplementary Figure 1). Culture medium was used as negative control. Supernatants were harvested for IFN-γ measurement by a standard sandwich ELISA, and the negative control values were subtracted from the final values reported. Shown are the survival of patients with pancreatic cancer in relation to IFN-γ production by peripheral blood T cells, based on the median cut-off values of IFN-γ levels (in pg/ml of cytokine concentration). ‘N.S.’ denotes a non-significant p value.

In an initial *in vitro* screen to identify immune recognition hotspots within the mesothelin molecule (pertaining to T-cell reactivity), the mesothelin_601-615_ peptide (MQEALSGTPCLLGPG) which is part of the mature mesothelin molecule expressed on the surface of pancreatic cancer cells, was identified as an immunodominant mesothelin peptide (Supplementary Figure 1 and Supplementary Table 2). The mesothelin_586-600_ peptide (LQGGIPNGYLVLDLS), which was also tested, resulted in strong cytokine production from T cells in the mesothelin screening experiments, and was thus used as an internal control for the activity of mesothelin_601-615_ in the present study with regard to survival of patients with pancreatic cancer. We observed that patient survival was associated with T-cell responses directed against the mesothelin_601-615_ peptide (p=0.006) (Figure 1G). A weaker but similar trend of patient survival associated with mesothelin601-615-specific IFN-γ production was also found without IL-21 conditioning albeit absence of statistical significance (Figure 1H). We did not observe a survival benefit for patients with pancreatic cancer in relation to T-cell responses directed to the mesothelin_586-600_ peptide, in the presence or absence of IL-21 conditioning (Figure 2A and 2B).

In the absence of IL-21 conditioning, the mesothelin_601-615_ peptide induced the strongest IFN-γ responses in peripheral blood of patients with pancreatic cancer, as compared to the precursor mesothelin peptide mix, the mature mesothelin peptide mix or the control mesothelin_586-600_ peptide (Supplementary Table 1). With IL-21 conditioning, the general pattern of the IFN-γ response to mesothelin_586-600_ was increased by more than 3-fold, while the response to mesothelin_601-615_ remained unchanged. With regard to differences between individual patients, we observed a general increase of IFN-γ production using the precursor mesothelin- or mature mesothelin stimulation, i.e. between a 2- and 6-fold increase of IFN-γ production in some samples. While there were some blood samples that exhibited a robust increase in IFN-γ production in the OKT3 group with IL-21 conditioning, other samples displayed total abrogation of cytokine responses after IL-21 conditioning. Interestingly, such a stark difference in IFN-γ responses, i.e. with or without IL-21 conditioning, was not seen among the individual peripheral blood samples when treated with medium alone (i.e. no specific stimulus was applied and therefore the spontaneous IFN-γ production was measured).

## DISCUSSION

Immunoreactivity studies gauging T-cell responses to mesothelin have been previously reported. Cytotoxic CD8+ T-cell responses to mesothelin-derived epitopes restricted by human leukocyte antigens (HLA)-A2, A3 and A24 have been described in surgically treated patients with pancreatic adenocarcinoma who received granulocyte-macrophage colony-stimulating factor-immunotherapy [23]. In addition, CD4+ T-cell mesothelin epitopes were also shown to induce IFN-γ production in peripheral blood from patients with pancreatic cancer [15]. In the present study, we show for the first time that IL-21 conditioning enhances mesothelin-specific T-cell responses, reflected by IFN-γ production in peripheral blood mononuclear cells, which correlates with increased survival of patients with pancreatic cancer who undergo surgery. The immunological recognition pattern, i.e. that only mature mesothelin (but not the MPF component of mesothelin), and particularly the immunodominant MQEALSGTPCLLGPG peptide (mesothelin aa 601-615) within the mature mesothelin is independently associated with survival, lends support that mesothelin-directed IFN-γ production provides a clinically relevant immune signature associated with improved clinical outcome.

IL-21 has been studied in preclinical models of cancer pertaining to the maintenance of antigen-specific CD8 T-cell responses [24], as well as in relation to the proliferation, survival and functionality of cancer-specific T cells isolated from patients with melanoma or pancreatic cancer [22, 25]. Additionally, activation of the IL-21/IL-21 receptor (IL-21R) pathway in T cells facilitates better control of human immunodeficiency virus (HIV) replication [26], while children with IL-21R deficiency are predisposed to opportunistic infections due to impaired cellular immunity [27].

More recently, exogenous IL-21 was shown to inhibit the expression of FoxP3, the cardinal transcription factor associated with regulatory T cells, in human CD4+ T cells following encounter with cancer cells – in a manner similar to TGF-β blockade [28]. We reported previously previously that a cytokine cocktail comprising IL-2, IL-15 and IL-21 selectively increases the population of central/effector memory tumor-reactive tumor-infiltrating lymphocytes (TIL) from pancreatic cancer as well as TIL from WHO grade 4 glioma after *ex vivo* expansion [21, 22]. Thus, IL-21 potentially selects for the long-term maintenance of a restricted, but highly efficient pool of antigen-specific T cells in the host – with a strong influence on disease control (cancer, chronic infections) and survival, a key observation with regard to the cellular therapy of cancer.

Importantly, the enhanced survival benefit seen among patients whose IFN-γ response to mesothelin_601-615_ was increased in the presence of IL-21 conditioning shows that peripheral blood immune cells recognizing this peptide are likely to express IL-21R on their surface. Interestingly, a study published in 2014 showed that mesothelin_601-615_ was exclusively recognized by circulating CD4+ T cells from healthy individuals, but not by T cells from patients with pancreatic cancer [15]. However, this was performed in the absence of IL-21 – conditioning, but with IL-2 supplementation for 9-10 days – which expands T-cells with a broad spectrum of TCR specificities, while IL-21 is more selective, particularly for memory CD8+ T cells.

Specific mesothelin epitopes, that are recognized by human CD8+ T cells, have been described before, although the amino acid residues 601-615 (as well as 586-600) are not among those previously reported [13, 29, 30]. It is thus possible that T cells with TCRs that are directed against mesothelin_601-615_ respond better to IL-21, and can be expanded to produce a measurable immunological readout in the presence of IL-21. Targeted immune responses to mesothelin_601-615_ may also bear clinical relevance in the diagnosis of patients with pancreatic cancer, since the use of a single peptide antigen may provide highly accurate immune-based readouts - in addition to being financially feasible for routine applications. T-cell reactivity to mesothelin_601-615_ could therefore be exploited in the context of immune monitoring and treatment outcomes of neoadjuvant therapy, chemotherapy as well as targeted immunotherapies in patients with cancer. Although we observed a significant increase in IFN-γ production in response to mesothelin_586-600_ after IL-21 conditioning, which was used as an internal control for comparison, we did not observe a correlation of cytokine response(s) to mesothelin_586-600_ with improved survival of patients with pancreatic cancer. It is possible that mesothelin_586-600_ does not encode a T-cell epitope for T cells that can recognize, kill or contain tumor cells and may therefore fail to evoke a clinically relevant and measurable immune reactivity *in vitro*.

Increased IFN-γ production, following OKT3 stimulation, was shown to be associated with decreased survival, a situation that may reflect non-productive inflammation and antigen-specific T-cell exhaustion [31]. The cell response to OKT3 is polyclonal due to activation of the TCR, and is therefore non-specific i.e. not related to a particular antigenic stimulus. Conversely, the antigen peptide mix represents a much more targeted cellular immune response i.e. by TCRs that recognize and respond to their cognate epitope(s). Since patients with pancreatic cancer are immunologically disadvantaged compared to healthy individuals, the OKT3 response allows for a more general overview of the functional T-cell status, and whether the T cells are able to respond to TCR stimulation at all.

We also observed that IL-21 conditioning of peripheral blood resulted in decreased IFN-γ production among circulating immune cells activated with the anti-human CD3 antibody (OKT3) – which may reflect the general immunological status of the cell-mediated immune response. This is plausible, since IL-21 can selectively inhibit IFN-γ production by memory Th17 cells and CD4+ Th1 effector cells and promote cytokine production by memory T-cell populations [32]. Furthermore, IL-21 can disrupt the activation and maturation of dendritic cells during an immune response, in addition to downregulation of MHC class II molecules [33]. This supports the case that memory CD8+ T cells are more likely to benefit from ‘IL-21 conditioning’, since they do not require APC-dependent activation, nor are they dependent on MHC class II-restricted antigen presentation.

Peripheral blood T cells from some patients produced more IFN-γ than others. This difference can be attributed to several factors: the extent of disease in each patient (at diagnosis, when blood was drawn), the general immunological status (in part reflected by the OKT3-directed cellular immune response), as well as the age of the patient. Nevertheless, we also observed that the background of IFN-γ production in peripheral blood i.e. in the absence of IL-21 conditioning, was rather low in the majority of patients. Thus, we hypothesize that IL-21 exhibits a biologically relevant and generally positive effect on circulating immune cells in patients with pancreatic cancer, associated with expansion of certain TCR specificities, such as specific mesothelin epitopes, described in this report.

## MATERIALS AND METHODS

### Patients

The Regional Ethics Review Board (Regionala etikprövningsnämnden) at Karolinska Institutet, Stockholm, Sweden, approved the study (diary number: 2013/977-31/1). Peripheral blood was obtained by venepuncture from 26 patients with pancreatic ductal adenocarcinoma (PDA) prior to starting on chemotherapy and undergoing surgery. A description of the patient cohort is provided in Table 1.

### Whole-blood assay

Whole blood assays (WBA) were performed by first diluting peripheral blood 5x with RPMI 1640 L-glutamine medium (ThermoFisher Scientific, Carlsbad, CA), followed by co-culture with 42 peptides spanning the full-length precursor mesothelin molecule before furin cleavage (19 peptides - MPF component; 23 peptides – mature mesothelin component), (Peptide and Elephants, Berlin, Germany) with or without conditioning with recombinant human IL-21 (10ng/ml) (Prospec, Rehovot, Israel) in 96-well plates (200μl diluted blood/well). The mature mesothelin epitope MQEALSGTPCLLGPG (mesothelin_601-615_) was also tested separately since we found this peptide to induce strong T-cell reactivity, defined by IFN-γ production, in peripheral blood obtained from eight patients with pancreatic cancer in an initial screen (Supplementary Information). In the same screen, another mesothelin peptide, LQGGIPNGYLVLDLS (mesothelin_586-600_), was also found to induce IFN-γ production, and was thus selected as an internal control for comparison in the present study. Other WBA antigens included in the study are listed in Table 2. Anti-human CD3 antibody (OKT3) was used as positive control while medium alone served as negative control. WBA plates were incubated for 7 days at 37°C with 5% CO_2_. Supernatants were harvested for IFN-γ detection by sandwich ELISA (Mabtech, Stockholm, Sweden). Final concentrations of antigen-specific IFN-γ production (pg/ml) were recorded after subtracting medium control values.

**Table 2:** List of antigens and controls used in the whole blood assay

Antigen	Type	Final concentration	Access NO.	Company
Anti-human CD3 antibody	antibody	30ng/ml	Clone:OKT3	Biolegend
Precursor mesothelin	peptide mix	1μg/peptide/ml	Q13421 (UniprotKB)	Peptides&Elephants
Mature mesothelin	peptide mix	1μg/peptide/ml	A12-114LB	Peptides&Elephants
MPF	peptide mix	1μg/peptide/ml	A12-114LB	Peptides&Elephants
Mesothelin _586-600_	single peptide	1μg/ml	Seq: LQGGIPNGYLVLDLS	Peptides&Elephants
Mesothelin _601-615_	single peptide	1μg/ml	Seq: MQEALSGTPCLLGPG	Peptides&Elephants

### Statistical analysis

Survival probability was evaluated using Kaplan-Meier curves and long-rank test. The cut-off median values of antigen-specific IFN-γ production were determined based on the intensity of response to each antigenic stimulus tested.

## CONCLUSIONS

IFN-γ responses in T-cells from peripheral blood, along with IL-21 conditioning and OKT3 stimulation, reflect pro-inflammatory cellular immune reactivity and decreased survival. Patients with subdued mesothelin-specific IFN-γ production appear to be at higher risk for cancer progression and would most likely benefit adapted monitoring and more targeted treatment strategies. Importantly, we are able to show that a single epitope (mesothelin_601-615_) can predict the probability of survival in patients with pancreatic cancer. This finding has major implications for novel diagnostic tests, whereby the use of a single epitope to measure clinically relevant immune responses in patients with pancreatic cancer increases feasibility, technical applicability and robustness. For patients undergoing immune checkpoint therapy i.e. with anti-programmed cell death 1 (PD-1), an increase in mesothelin_601-615_-specific T cells could be indicative of improved clinical outcomes, although this needs to be formally evaluated. Preclinical (murine model of human pancreatic ductal adenocarcinoma) evaluation of a TCR-engineered T-cell product specific for mesothelin_406-414_ showed increased immune cell infiltration into the tumor site, coupled with improved survival of mice [12]. Furthermore, mesothelin-directed chimeric antigen receptors (CAR) are also in clinical trials for various cancers [34]. A clinical trial in its recruitment phase for investigating the safety, efficacy and tolerability of anti-mesothelin antibody (BMS-986148) in conjunction with anti-PD-1 (nivolumab) in patients with advanced solid tumors including patients with pancreatic cancer (NCT02341625) is underway. From an immunotherapy viewpoint, the absence of naturally-occurring mesothelin-directed cellular immune responses in the patient may be augmented with supportive strategies, if an appropriate T-cell repertoire exists that can be measured *in vitro* by expansion of mesothelin-specific T cells, e.g. using adjunctive IL-21 conditioning. Our findings call for a more tailored follow-up of patients with pancreatic cancer and encourage host-directed therapies for patients with advanced gastrointestinal malignancies.

## SUPPLEMENTARY MATERIALS FIGURE AND TABLES


