# Supplementary material for: Prediction of improved survival in patients with pancreatic cancer via IL-21 enhanced detection of mesothelin epitope-reactive T-cell responses
Source: Oncotarget. 2018 Apr 27;9(32):22451–9. doi: 10.18632/oncotarget.25121 (PMC5976477; doi:10.18632/oncotarget.25121)
Supplement: Supplementary file 1 [file oncotarget-09-22451-s001.pdf]

Prediction of improved survival in patients with pancreatic cancer via IL-21 enhanced detection of mesothelin epitope-reactive T-cell responses

SUPPLEMENTARY MATERIALS

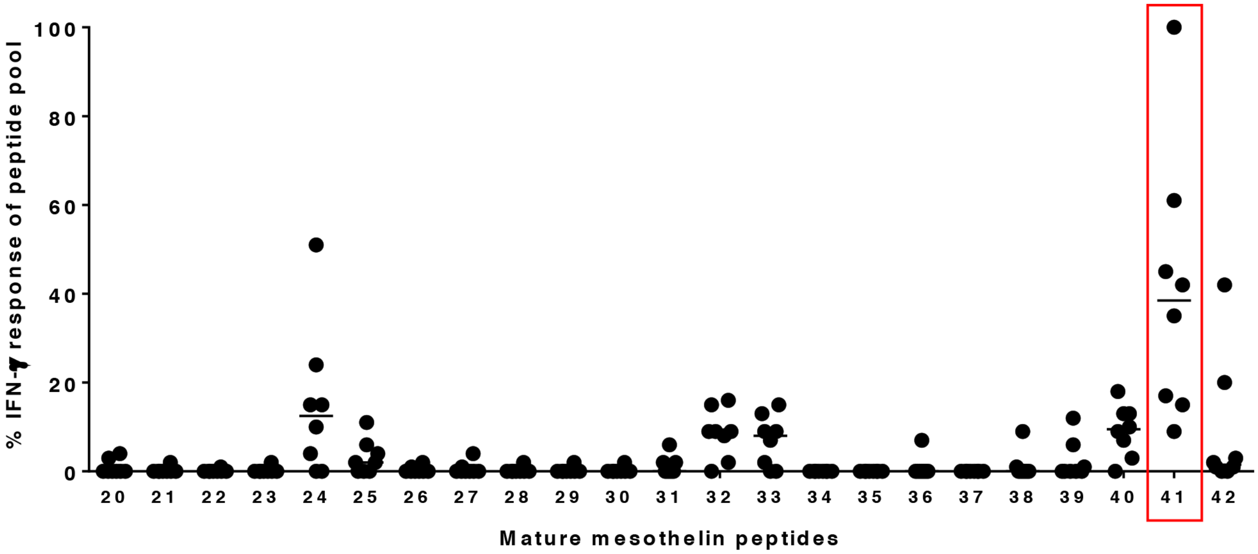

Supplementary Figure 1: Mature mesothelin peptide mapping. Based on data presented in Supplementary Table 2, the percentage of peptide-specific production of IFN-γ relative to the entire sequence of mature mesothelin was calculated as follows: (*IFN-γ response to individual peptide / Sum of IFN-γ response to all peptides) x 100*. This figure presents the immune recognition hotspots within the mature mesothelin molecule, and the potential T-cell epitopes represented by IFN-γ production. IFN-γ responses to peptide 41 (mesothelin$_{601-615}$) is highlighted in a red box, and displays the most robust responses. Response to peptide 40 (mesothelin$_{586-600}$) is also shown, and was selected as a control peptide to measure IFN-γ response in PBMCs from patients with pancreatic cancer, followed by testing the association of peptide- directed cytokine responses and patient survival.

Supplementary Table 1: Individual IFN-γ responses to various stimulation conditions in the whole-blood assays

Patient ID (#)	Without IL-21						With IL-21					
	OKT3	Precursor mesothelin	Mature mesothelin	Mesothelin 601-615	Mesothelin 586-600	Medium only	OKT3	Precursor mesothelin	Mature mesothelin	Mesothelin 601-615	Mesothelin 586-600	Medium only
2	2480,71	18,97	NA	NA	NA	313,26	2106,29	0,00	NA	NA	NA	447,45
5	417,80	515,96	NA	NA	NA	0,00	0,00	223,52	NA	NA	NA	0,00
7	18,68	42,11	NA	NA	NA	0,00	0,00	43,13	NA	NA	NA	0,00
19	852,17	275,54	742,01	NA	NA	0,00	0,00	445,05	131,49	NA	NA	0,00
24	664,49	263,19	636,79	NA	NA	0,00	703,68	387,70	657,42	NA	NA	0,00
31	3667,00	18,78	1,05	NA	NA	31,13	3667,00	40,44	26,24	NA	NA	48,91
34	1397,18	0,00	0,00	NA	NA	37,83	3667,00	14,96	0,00	NA	NA	38,16
41	1086,23	2334,09	581,74	NA	NA	0,00	0,00	2257,03	1028,97	NA	NA	0,00
50	0,00	0,01	0,01	NA	NA	40,53	95,69	3,90	0,00	0,00	0,00	42,58
51	100,53	11,38	0,00	NA	NA	15,50	32,76	9,20	4,08	0,00	0,00	14,93
59	3667,00	6,86	0,00	0,00	3667,00	0,00	3667,00	10,09	0,00	0,00	3667,00	0,00
62	40,30	6,50	4,30	8,70	4,30	9,85	17,00	9,60	0,70	146,50	2,90	64,04
63	366,50	0,00	0,00	1,20	1,20	12,92	3667,00	4,40	0,00	5,50	2,20	7,09
68	663,02	100,30	22,31	83,82	116,87	251,29	351,70	94,69	34,29	118,77	68,94	286,71
72	2267,58	2,38	0,00	1,59	0,00	5,05	1355,94	21,16	0,00	6,54	2,90	3,35
74	192,93	4,67	0,00	5,45	5,45	9,80	25,07	10,60	3,02	16,68	8,32	9,84
76	115,84	0,99	0,00	1,99	0,99	2,37	228,94	5,76	0,00	175,76	0,96	2,87
81	48,41	3,28	1,09	4,37	5,46	0,00	58,39	8,74	1,46	10,20	5,83	0,00
87	328,20	223,53	29,72	119,65	203,36	246,11	17,71	248,76	46,99	152,89	103,34	314,65
99	209,70	0,00	0,00	0,00	0,00	0,00	279,26	0,00	0,00	0,00	0,00	0,00
104	151,00	3,80	0,00	5,33	3,04	0,00	NA	NA	NA	NA	NA	NA
107	17,40	21,50	0,00	18,43	8,19	22,08	14,56	29,14	2,24	30,26	31,38	20,26
114	0,16	0,01	0,04	0,02	0,00	2,20	55,03	19,90	0,00	1,77	5,93	33,44
116	106,97	23,70	11,84	209,97	40,31	0,00	137,01	128,65	41,30	417,36	24,76	0,00
117	3,57	1,43	0,00	0,71	2,14	22,99	0,00	14,92	0,00	11,40	7,01	20,90
120	30,72	169,98	4,71	382,95	177,28	0,00	0,00	533,24	73,97	1903,23	56,66	0,00

OKT3 (anti-human CD3), precursor mesothelin peptide mix, mature mesothelin peptide mix, mesothelin601-615 and mesothelin586-600 with and without IL-21 conditioning of peripheral blood from patients with pancreatic cancer. IFN-γ production in the medium control (background IFN-γ production) has been removed from the responses to the OKT3 and the respective antigen stimulation conditions.

Supplementary Table 2: IFN-γ responses to mature mesothelin peptide pool

		IFN-γ production (pg/ml)							
	**Sequence**	**Patient 1**	**Patient 2**	**Patient 3**	**Patient 4**	**Patient 5**	**Patient 6**	**Patient 7**	**Patient 8**
Mesothelin-20	RTILRPRFRREVEKT	0	0	0	1	0	0	1	3
Mesothelin-21	ACPSGKKAREIDESL	0	0	0	0	0	0	1	0
Mesothelin-22	IFYKKWELEACVDAA	0	0	0	5	0	0	0	0
Mesothelin-23	LLATQMDRVNAIPFT	0	0	0	5	0	0	0	0
Mesothelin-24	YEQLDVLKHKLDELY	0	0	68	52	9	283	1	56
Mesothelin-25	PQGYPESVIQHLGYL	0	0	2	8	1	210	1	6
Mesothelin-26	FLKMSPEDIRKWNVT	0	0	0	0	0	12	0	2
Mesothelin-27	SLETLKALLEVNKGH	0	0	0	0	0	0	1	1
Mesothelin-28	EMSPQAPRRPLPQVA	0	0	0	0	0	0	1	0
Mesothelin-29	TLIDRFVKGRGQLDK	0	0	0	0	0	0	1	0
Mesothelin-30	DTLDTLTAFYPGYLC	0	0	0	0	0	0	1	0
Mesothelin-31	SLSPEELSSVPPSSI	0	0	0	8	1	0	2	0
Mesothelin-32	WAVRPQDLDTCDPRQ	0	1	59	56	5	172	4	10
Mesothelin-33	LDVLYPKARLAFQNM	0	14	61	7	3	257	0	8
Mesothelin-34	NGSEYFVKIQSFLGG	0	0	0	0	0	0	0	0
Mesothelin-35	APTEDLKALSQQNVS	0	0	0	0	0	0	0	0
Mesothelin-36	MDLATFMKLRTDAVL	0	0	0	0	0	127	0	0
Mesothelin-37	PLTVAEVQKLLGPHV	0	0	0	0	0	0	0	0
Mesothelin-38	EGLKAEERHRPVRDW	0	0	0	0	0	171	0	1
Mesothelin-39	ILRQRQDDLDTLGLG	0	0	0	22	0	0	5	1
Mesothelin-40	LQGGIPNGYLVLDLS	0	6	90	37	5	345	1	10
Mesothelin-41	MQEALSGTPCLLGPG	161	33	434	149	5	328	18	10
Mesothelin-42	PVLTVLALLLASTLA	0	39	0	7	7	25	1	1
	**Total IFN-γ production / patient**	**161**	**94**	**714**	**357**	**36**	**1930**	**40**	**110**

Non-overlapping peptides (15-mer) spanning the length of the mature mesothelin sequence were co-cultured with whole blood in a 7-day whole-blood assay to gauge for IFN-γ production. Supernatants were harvested for IFN-γ detection by sandwich ELISA. Negative control values (medium control) were subtracted from the result prior to reporting. The peptide sequences from the mature mesothelin are listed.
